# Comprehensive analysis illustrating the role of PANoptosis-related genes in lung cancer based on bioinformatic algorithms and experiments

**DOI:** 10.3389/fphar.2023.1115221

**Published:** 2023-02-16

**Authors:** Shiyou Wei, Zhigang Chen, Xinyu Ling, Wentian Zhang, Lei Jiang

**Affiliations:** ^1^ Department of Anesthesiology, Shanghai Pulmonary Hospital, School of Medicine, Tongji University, Shanghai, China; ^2^ Department of Thoracic Surgery, Shanghai Pulmonary Hospital, School of Medicine, Tongji University, Shanghai, China

**Keywords:** PANoptosis, FADD, lung cancer, immunotherapy, proliferation

## Abstract

**Background:** Recently, PANoptosis has aroused the interest of researchers for its role in cancers. However, the studies that investigated PANoptosis in lung cancer are still few.

**Methods:** The public data were mainly collected from The Cancer Genome Atlas (TCGA) and Gene Expression Omnibus database. R software was utilized for the analysis of public data. Quantitative real-time (qRT) polymerase chain reaction (PCR) was used to measure the RNA level of FADD. The cell proliferation ability was evaluated using the CCK8, colony formation, and 5-ethynyl-2′-deoxyuridine (EdU) assays. Western blot was used to detect the protein level of specific molecules. Flow cytometry analysis and TUNEL staining were used to evaluate cell apoptosis.

**Results:** In our study, we collected the PANoptosis-related genes from previous studies. Through series analysis, we identified the FADD, an adaptor of PANoptosis and apoptosis, for further analysis. Results showed that FADD is one of the prominent risk factors in lung cancer, mainly localized in nucleoplasm and cytosol. We next performed immune infiltration analysis and biological enrichment to illustrate the underlying cause of FADD in lung cancer. Subsequently, we discovered that the patients with a high level of FADD might respond worse to immunotherapy but better to AICAR, bortezomib, docetaxel, and gemcitabine. *In vitro* experiments indicated that inhibiting FADD could reduce significantly the ability of cancerous lung cells to proliferate. Meanwhile, we found that the knockdown of FADD promotes the apoptosis and pyroptosis. Ultimately, a prognosis signature was identified based on the FADD-regulated genes, which showed satisfactory prediction efficiency on patients with lung cancer.

**Conclusion:** Our result can provide a novel direction for future studies focused on the role of PANoptosis in lung cancer.

## Introduction

According to International Agency for Research on Cancer (IARC), lung cancer has the highest mortality rate and the second-highest incidence rate among all cancers (Sung et al., 2021). About 2 million people are annually diagnosed with lung cancer globally, and over 85% of them are diagnosed with the most prevalent pathological subtype, i.e., non-small cell lung cancer (NSCLC) (Molina et al., 2008; Sung et al., 2021). As a result of current advances in diagnostic techniques and treatment protocols, such as the widespread availability of chest computed tomography and the clinical application of targeted therapies and immunotherapy, survival rates have improved for some patients (Aberle et al., 2011; Reck et al., 2022; Tan and Tan, 2022), but there are still 1.76 million lung cancer deaths worldwide each year (Sung et al., 2021). Therefore, it is necessary to conduct further research on lung cancer.

Cell death is a widespread phenomenon in living organisms and is essential for maintaining homeostasis in the organism. Programmed cell death (PCD) is a genetically determined mode of active cell death that includes pyroptosis, apoptosis, and necroptosis, which was tightly correlated with both homeostasis and disease (Malireddi et al., 2021). The crosstalk in pyroptosis, apoptosis, and necroptosis pathways led to the establishment of the concept of PANoptosis, defined as an inflammatory PCD pathway regulated by the PANoptosome complex with key features of pyroptosis, apoptosis, and/or necroptosis that cannot be accounted for by any of these PCD pathways alone (Wang et al., 2022a). Li et al. found that the complex formed by AIM2, pyrin and ZBP1 could drive PANoptosis and host defense (Lee et al., 2021). Recently, researchers began to pay attention to the role of PANoptosis in diseases. Karki et al. noticed that the ADAR1 could restrict the PANoptosis and ZBP1-mediated immune response to facilitate tumorigenesis (Karki et al., 2021). Karki et al. revealed that the IFN therapeutic efficacy could be disrupted by PANoptosis, ZBP1-dependent inflammation, and cytokine storm during COVID-19 infection (Karki et al., 2022). Wang’s *in vivo* experiments found that ferroptosis occurring in the tumor can be regulated by CD8^+^ T cells, suggesting that ferroptosis can be involved in anti-tumor immunity (Wang et al., 2019a). Chemotherapeutic agents can inhibit proliferation and metastasis by inducing pyroptosis of lung cancer, such as paclitaxel and cisplatin (Li et al., 2019). PANoptosis, with critical features of pyroptosis, necroptosis, and apoptosis, has become the focus of recent cancer research (Wang and Kanneganti, 2021). A growing number of studies report PANoptosis involvement in multiple cancer biological processes, like chemotherapy resistance in colorectal cancer and immunotherapy response in gastric cancer (Pan et al., 2022a; Lin et al., 2022). Therefore, research on PANoptosis in tumors has a broad clinical perspective.

The rapid development of next-sequence technology help researchers better understands diseases (Yin et al., 2020; Wei et al., 2021; Gu et al., 2022). In our study, we collected the PANoptosis-related genes from previous studies. Through series analysis, we identified the FADD, an adaptor of PANoptosis and apoptosis, for further analysis. Results showed that FADD is a risk factor for lung cancer and is mainly localized in nucleoplasm and cytosol. We next performed biological enrichment and analysis of immune infiltration to illustrate the possible effect of FADD on lung cancer. Subsequently, the results showed that high levels of FADD in patients may lead to reduced immunotherapy efficacy but help in treatment with AICAR, bortezomib, docetaxel, and gemcitabine. Further, the inhibition of FADD by *in vitro* experiments reduced the proliferation capacity of lung cancer cells. Ultimately, a prognosis signature was identified based on the FADD-regulated genes, which showed satisfactory prediction efficiency on the patient’s OS in internal and external validation cohorts and the training cohort.

## Methods

### Public data collection

The public data were mainly collected from Gene Expression Omnibus (GEO) and the Cancer Genome Atlas (TCGA) databases. Extensive expression profiles and associated clinical data were obtained from the TCGA-GDC program and subsequently incorporated into the TCGA database (TCGA-LUSC and TCGA-LUAD projects). The “STAR-Counts” format was used for each patient’s expression file, whereas the “bcr xml” format was used for clinical information. The perl and R codes of the authors were utilized for data collation. The probe annotation used the GRCh38.p13.gtf, which can be downloaded from the Ensembl website and corresponds to the human genomic reference file. The genes with a median value <0.1 were removed. Meanwhile, the gene expression value was converted into log2 to reduce the impact of extreme value. For the GEO database, the GSE30219 was selected, whose annotation platform is GPL570 [HG-U133_Plus_2] Affymetrix Human Genome U133 Plus 2.0 Array. Patients’ expression profiles and clinical information in GSE30219 were downloaded from the link “Series Matrix File(s).” Before analysis, the expression profile data of GSE30219 were pro-precessed using the limma package, including missing value completion, correction, normalization, and so on. Human Protein Altas (HPA) project can provide representative immunohistochemical data and corresponding protein level evaluation of encoding genes. The immunohistochemical image of FADD and its subcellular localization information was obtained from the HPA website through online searching. The expression data of FADD in pan-cancer was downloaded from the website USCS-Xena (https://xenabrowser.net/datapages/). The limma package was utilized to identify differentially expressed genes (DEGs) (Ritchie et al., 2015). For DEGs analysis between patients with high and low FADD expression, the threshold was set as |logFC| > 0.6 and *p*-value <0.05.

### Collection of the molecules involved in the PANoptosis process

The genes involved in the PANoptosis process were collected from previous studies, including ADAR, MEFV, AIM2, NLRP1, NLRC4, NLRP9, NLRP3, ZBP1, TNFRSF1A, PYCARD, FADD, CASP10, CASP1, CASP2, CASP12, CASP4, CASP3, CASP6, CASP5, CASP7, DFNA5, CASP8, MLKL, GSDMD, RIPK3, RIPK1, NAIP, TNF, NLRP6, GSDMA, GSDMC, GSDMB, APAF1, BAK, BAX, DIABLO, DCN, CASP9, and FAS (Wang and Kanneganti, 2021).

### Protein-protein interaction (PPI) network

The STRING database (https://cn.string-db.org/) was used to construct the PPI network. Herein, the “Organism” used was “Homo,” and the “minimum required interaction score” was “medium confidence.” Cytoscape was used for visualization of the PPI network. Cytoscape software was used for visualization and clueGO plug-in was used for the biological enrichment analysis of nodes (Shannon et al., 2003).

### Clinical and prognosis analysis

The prognosis difference between the two groups was compared using the KM (Kaplan-Meier) survival curves and *p* < 0.05 was regarded as statistically significant. We used univariate and multivariate logistic regression to identify variables that can independently affect patients’ survival (*p* < 0.05 was regarded as statistically significant).

### Identification of prognosis signature

Using univariate cox-regression, prognosis-related genes were determined for use as the input genes. Subsequently, the LASSO regression analysis was applied to reduce data dimension and identify optimization variables. Finally, the multivariate Cox regression was utilized for signature identification of prognosis with the following equation “Risk score = (Gene_A_ × *A*) + (Gene_B_ × *B*) + (Gene_C_ × *C*) + (…) + (Gene_N_ × *N*), where *A-N* are the coefficients” (Wu et al., 2021). KM and receiver operating characteristic (ROC) curves were utilized to evaluate the prognosis prediction efficacy. For TCGA cohort, the patients were randomly divided into training and validation cohort in proportion of 1:1. The GSE30219 was selecte as the external validation corhort.

### Nomogram plot

The rms R package was utilized to establish a nomogram plot combining clinical parameters and the risk score. To evaluate the fit between the actual survival and nomogram-predicted survival, the calibration and decision curve analysis (DCA) were generated.

### Biological enrichment analysis

Gene Ontology (GO) analysis was conducted using the clusterprofiler software (Yu et al., 2012). The biological variations between the two groups were determined using Gene Set Enrichment Analysis (GSEA) on the basis of the set pathway reference set (Subramanian et al., 2005). The terms with *p* < 0.05 were regarded as statistically significant.

### Immune infiltration analysis

On the basis of gene expression profile, the tumor immune microenvironment of patients with lung cancer was quantified using multiple algorithms, including CIBERSORT, EPIC, MCPCOUNTER, QUANTISEQ, TIMER, and XCELL algorithms (Chen et al., 2018; Plattner et al., 2020; Racle and Gfeller, 2020).

### Immunotherapy and drug sensitivity analysis

Patient immunotherapy responses were assessed utilizing the TIDE (Tumor Immune Dysfunction and Exclusion) program (Fu et al., 2020). Each patient was given a TIDE score, with scores >0 indicating poor immunotherapy response (or responders) and a score <0 indicating no success (non-responders). Drug sensitivity testing was performed using the Genomics of Drug Sensitivity in Cancer (GDSC) database (Yang et al., 2013).

### Cell culture

The BEAS-2B, A549, H1299, and H441 cells were routinely stored in the laboratory. The cell line authentication was carried out using STR detection. They were cultured in the 10% heat-inactivated fetal bovine serum at 37°C with 5% CO_2_. Depending on the confluence of the cells, the cells were passaged every 3-4 days.

### Quantitative real-time (qRT) PCR

Utilizing an RNA extraction kit (TaKaRa Bio), total RNA was isolated. Then, cDNA was synthesized by reverse-transcribing utilizing a high-capacity cDNA Reverse Transcription Kit. Using TB Green Premix Ex Taq, we performed RT-PCR to measure the levels of mRNA expression levels. The PCR primers were synthesized by Tsingke (Beijing, China). The sequence of primers was as follows: FADD, forward, 5′-GTG​GCT​GAC​CTG​GTA​CAA​GAG-3′, reverse, 5′-GGT​AGA​TGC​GTC​TGA​GTT​CCA​T-3′; GAPDH, forward, 5′-TTG​TCT​CCT​GCG​ACT​TCA​ACA​G-3′, revers, 5′-GGT​CTG​GGA​TGG​AAA​TTG​TGA​G-3′.

### Cell transfection

GenePharma Co., Ltd. generated both FADD knockdown plasmids and control plasmids. H1299 (5 × 10^5^) cells on a 6-well plate were plated and cultured at 37°C overnight using RPMI-1640. These cells were incubated in serum-free Gibco™-OptiMEM (from Thermo Fisher Scientific, Inc.) for 6 h at 37°C after being treated with shRNA and Lipofectamine^®^2000 Reagent (from Invitrogen, Thermo Fisher Scientific, Inc.). The cells were then added with Fresh RPMI-1640 containing 10% FBS. For the effect of FADD on the proliferation of H1299, the concentrations of FADD siRNA were 0, 10, 25, 50, 100, and 200 nM, and the concentrations of the total shRNA were made up to 200 nM with NC shRNA for those transfections with a FADD shRNA concentration <200 nM. After 48 h of shRNA transfection, H1299 cells were treated with Adriamycin to observe the effect of FADD on the cells’ drug resistance, where the concentration of FADD shRNA used was 100 nM.

### Cell proliferation assay

The assessment of cell proliferation ability was conducted using the CCK8, colony formation, and 5-ethynyl-2′-deoxyuridine (EdU) assays. To perform the CCK8 assay, cell culture media was introduced with CCK-8 reagent at a mixed ratio of 1:10 (Beyotime, Shanghai, China). Cell proliferation was assessed by measuring absorbance at wavelength 450 nm immediately after incubation at 37°C for 2 h. Colony formation assays involve cultivating lentivirus-infected cells for 14 days after seeding 2000 of them into six-well plates. After 10 min of treatment in 4% paraformaldehyde, the cells were stained for 30 min with crystal violet (from Beyotime, Shanghai, China). For the EdU assay, cells were inoculated on coverslips of 12-well plates at suitable cell numbers. The following day, EdU working solution was co-cultured with the cells for 2 h. Subsequent fixation and staining were performed according to the EdU (from Beyotime, Shanghai-China) instructions and photographed by fluorescent microscope.

### Flow cytometry analysis

According to the manufacturer’s instructions, the cells were stained with annexin V/fluorescein isothiocyanate (FITC) kit (BD Biosciences, Franklin Lakes, NJ, United States). In short, H299 cells were cultured in 6-well plates. We collected cells and added 100 μL combined buffer and 5 μL fluorescein isothiocyanate (FITC) labeled annexin V (20 μ G/mL) and incubate at room temperature in the dark for 15 min. Then, the mixture were added 5 μL propidium iodide (PI, 50 μ G/mL) and incubated in the dark for 5 min. After that, we added 400 μL combined with buffer solution and immediately performed FACscan for quantitative detection by flow cytometry.

### TUNEL staining

TUNEL apoptosis detection kit (Suzhou Yuheng Biotechnology Co., Ltd.) was used to detect cell apoptosis. Cells (1 × 10^5^) were fixed with 4% polyformaldehyde, and 0.2% Triton X-100 was used for penetration. We then added 50 μL of TUNEL reaction mixture and incubated it in dark at 37°C for 60 min, and then used DAPI solution to re-stain the nucleus. The cells were observed under fluorescence microscope (Zeiss) and the apoptosis rate was calculated by ImageJ software.

### Western blot

Cells were lysed in pre-cooled RIPA lysis buffer (Beyotime) with freshly added protease inhibitor mixture (Hoffman-La Roche Ltd., Basel, Switzerland). The protein concentration was determined by the Dioctanoic acid determination kit (Thermo Fisher Scientific). The same amount of protein was separated by SDS-PAGE and transferred to PVDF membrane. The membrane and the first antibody (Bcl-2, Bax, pro-caspase-1, NLRP3, GAPDH) were incubated at 4°C overnight. After incubation with HRP-labeled secondary antibody at room temperature for 2 h, the protein bands were analyzed using ECL Hypersensitive Chemiluminescence Kit (Beyotime).

### Statistical analysis

All the analysis was performed using the R, SPSS, GraphPad Prism eight and ImageJ software. Statistically significant was reached when *p* < 0.05. The *t*-test was utilized for assessing normally distributed data. The Mann–Whitney-U test was utilized for assessing non-normally distributed data.

## Results

### Role of PANoptosis-related genes in lung cancer

**FIGURE 1:**
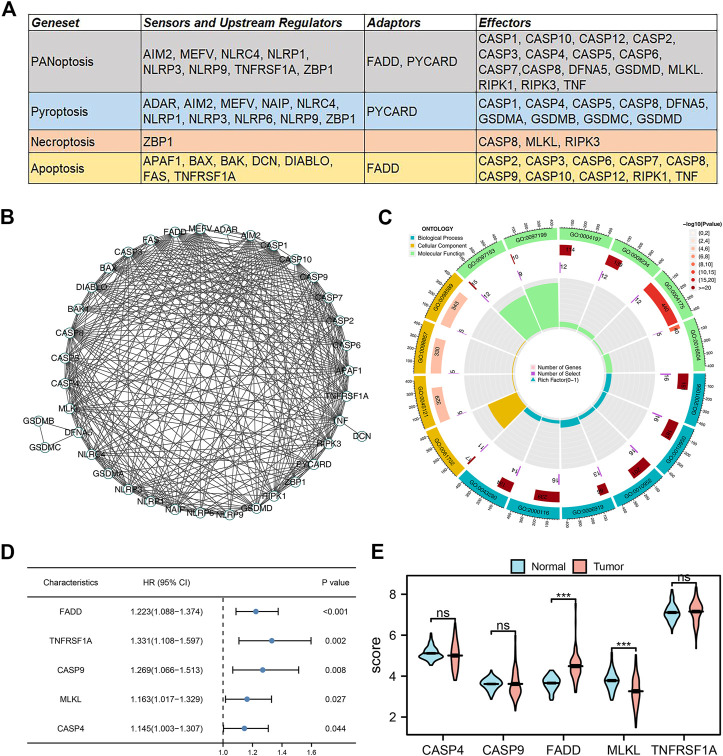
Identification of PANoptosis-related genes in lung cancer. Notes: **(A)** The PANoptosis-related genes collected from previous studies; **(B)** PPI network of these PANoptosis-related genes; **(C)** GO analysis of these PANoptosis-related genes; **(D)** Univariate Cox regression of these PANoptosis-related genes; **(E)** The expression level of CASP4, CASP9, FADD, MLKL and TNFRSF1A in tumor and normal tissue.

The flow chart of whole study was shown in Supplementary Figure S1. According to the previous studies, we collected the molecules involved in the PANoptosis, which is shown in Figure 1A. These genes are involved in PANoptosis, Pyroptosis, Necroptosis, and Apoptosis. The PPI network of these PANoptosis-related genes is depicted in Figure 1B. GO analysis indicated that these genes were mainly involved in the execution phase of apoptosis (GO:0097199), cysteine-type endopeptidase activity (GO:0004197), endopeptidase activity (GO:0004175), cysteine-type peptidase activity (GO:0008234), peptidase activator activity (GO:0016504), positive regulation of cysteine-type endopeptidase activity (GO:2001056), positive regulation of endopeptidase activity (GO:0010950), positive regulation of peptidase activity (GO:0010952), activation of cysteine-type endopeptidase activity involved in the apoptotic process (GO:0006919), regulation of cysteine-type endopeptidase activity (GO:2000116), positive regulation of cysteine-type endopeptidase activity involved in the apoptotic process (GO:0043280), inflammasome complex (GO:0061702), membrane raft (GO:0045121), membrane microdomain (GO:0098857), membrane region (GO:0098589), cysteine-type endopeptidase activity involved in the apoptotic process (GO:0097153) (Figure 1C). Univariate Cox regression analysis was applied to identify the genes significantly correlated with the patient’s survival (Supplementary Material S1). The results indicated that the genes FADD, TNFRSF1A, CASP9, MLKL, and CASP4 were the risk factor for lung cancer patients (Figure 1D, *p* < 0.05). Among these genes, FADD had the smallest *p*-value and aroused our interest. We noticed that FADD was also highly expressed in lung cancer tissue compared to paracancerous tissue (Figure 1E). Meanwhile, it is also an adaptor for PANoptosis and Apoptosis processes. Therefore, the FADD was selected for further analysis.

### Clinical role of FADD in lung cancer

**FIGURE 2:**
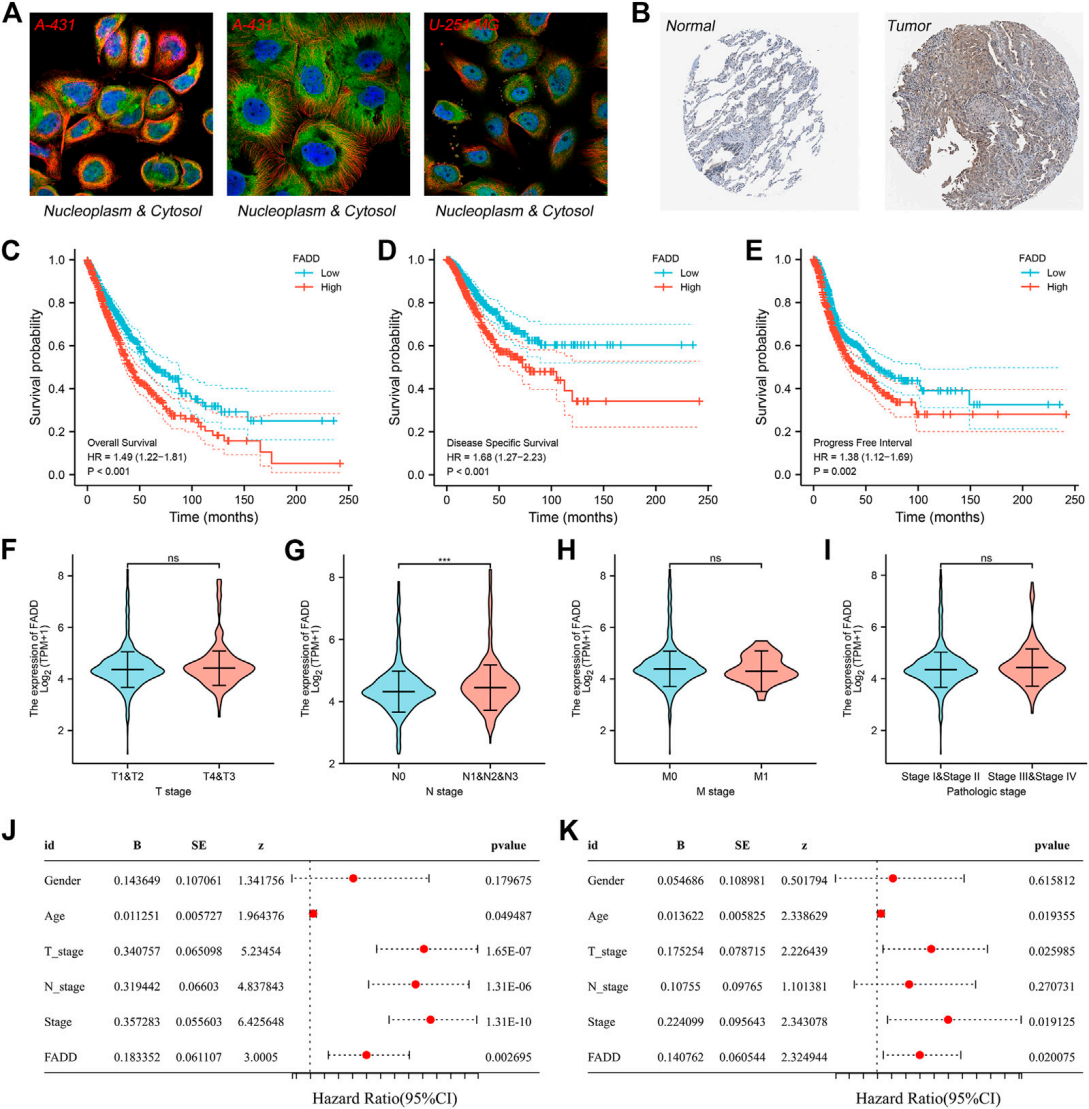
Clinical role of FADD in lung cancer. Notes: **(A)** Subcellular localization of FADD; **(B)** Immunohistochemistry image of FADD in lung cancer tumor and normal tissue; **(C)** The OS difference in high- and low-FADD expression patients; **(D)** The difference in DSS among patients having high and low FADD expression; **(E)** The difference in PFI among patients having high and low FADD expression; (**F-I)**: Clinical correlation of FADD; (**J-K)**: Univariate and multivariate analysis of FADD.

Subsequently, using the HPA database, we analyzed the subcellular location of FADD. The result indicated that FADD was primarily localized in the nucleoplasm and cytosol based on the A-431 and U-251 MG cell lines (Figure 2A). Representative immunohistochemistry images obtained from the HPA database indicated a higher protein level of FADD in the lung cancer tissue (Figure 2B). KM survival curves demonstrated that patients having higher-level FADD expression tend to have a poor prognosis performance in terms of disease-free survival (DSS), overall survival (OS), and progression-free survival (PFI) (Figures 2C–E). The prognosis prediction efficiency of FADD in OS was shown in Supplementary Figure S2. Next, we assessed the correlation between FADD and the clinical stage. The result depicted that the FADD could be related to the worse N classification but not the T and M classifications (Figures 2F–I). Statistical univariate and multivariate regression analyses indicated that FADD is an independent factor of risk in the prognosis of a lung cancer patient (Figures 2J, K, Univariate, *p* = 0.002, Multivariate, *p* = 0.02). Supplementary Figure S3 depicts the FADD expression pattern of the pan-cancer.

### Biological investigation of FADD in lung cancer

**FIGURE 3:**
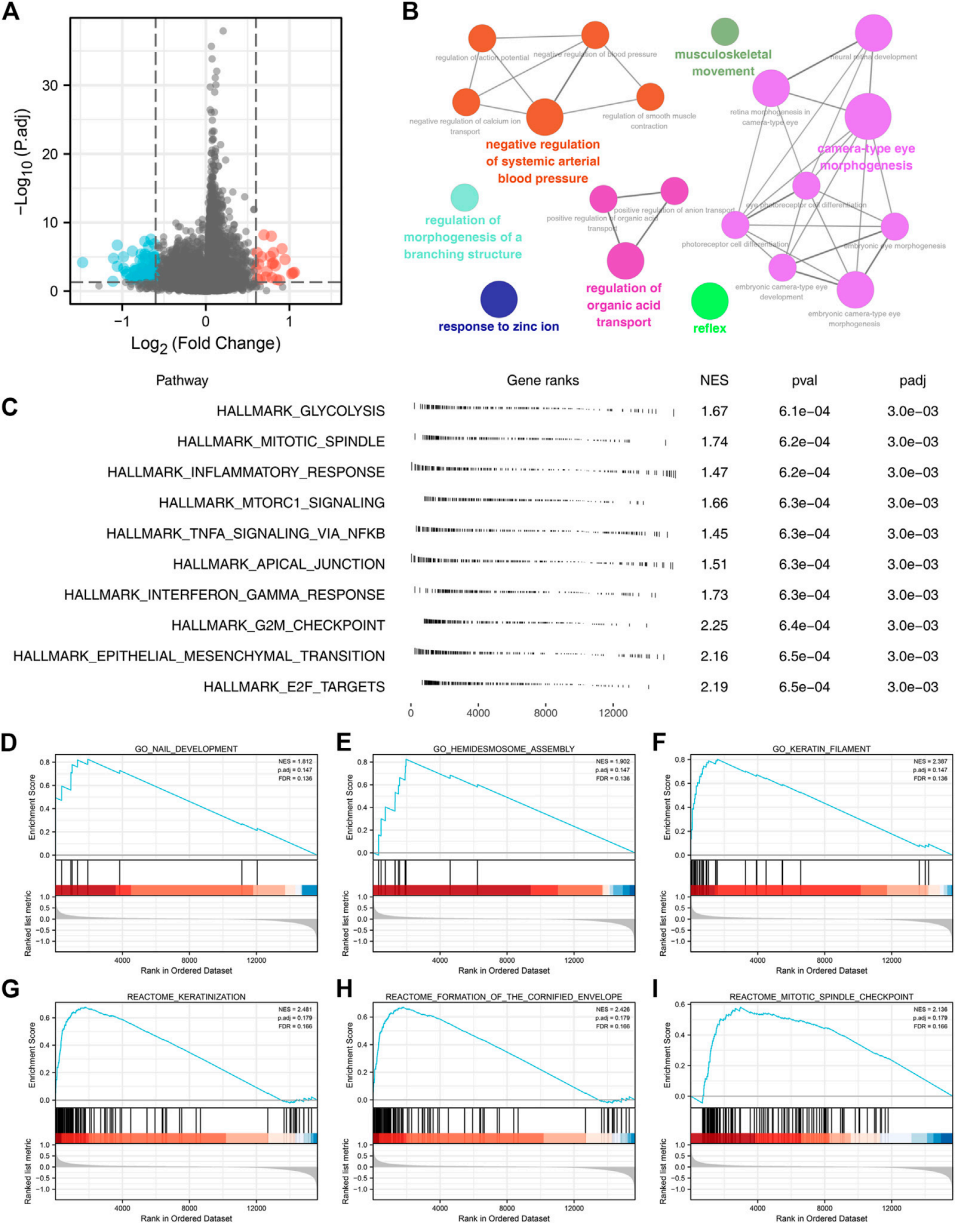
Biological investigation of FADD in lung cancer. Notes: **(A)** DEGs analysis of high- and low-FADD patients; **(B)** ClueGO analysis; **(C)** GSEA analysis of FADD; (**D-F)**: GSEA analysis based on GO gene set; (**G-I)**: GSEA analysis based on REACTOME gene set.

Then, we evaluated the biological role of FADD in lung cancer. Firstly, we conducted the DEGs analysis in high- and low-FADD expression patients with criteria of *p* < 0.05 and log FC > 0.6 (Figure 3A). ClueGO assessment indicated that these DEGs were primarily enriched in musculoskeletal movement, negative regulation in systemic arterial BP, morphogenesis regulation of a branching structure, response to zinc ion, regulation of organic acid transport, reflex, and camera-type eye morphogenesis (Figure 3B). GSEA analysis based on the Hallmark gene set indicated that the pathways of glycolysis, mitotic spindle, inflammatory response, mTORC1 signaling, interferon-gamma response, apical junction, TNFA signaling through NFKB, G2M check-point, epithelial-mesenchymal transition (EMT), and E2F target were activated in patients with high FADD expression (Figure 3C). Also, for the GSEA analysis based on GO and REACTOME gene sets, we noticed that the terms of nail development, hemidesmosome assembly, keratin filament, keratinization, formation of the cornified envelope, mitotic spindle check-point were enriched in (Figures 3D–I).

### Immune-related analysis

**FIGURE 4:**
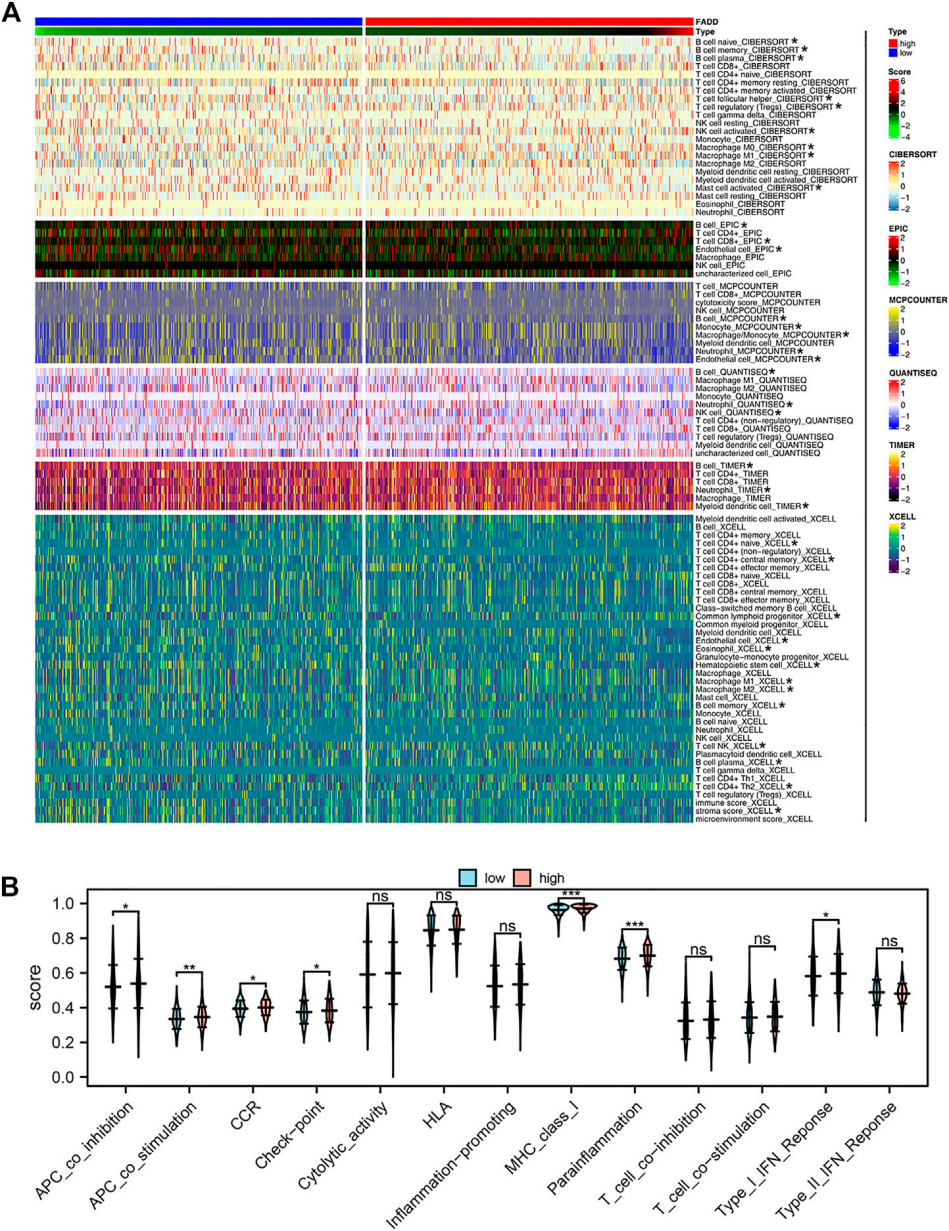
Effect of FADD on immune infiltration and function. Notes: **(A)** The correlation between quantified immune cells and FADD; **(B)** The activity of multiple immune functions in high- and low-FADD expression patients.

After that, we looked at how FADD affected the immune microenvironment of lung cancer. The result indicated that FADD could remarkably affect specific immune cells, including naïve B cells, plasma B cells, memory B cells, follicular helper T cells, activated NK cells, Tregs, activated mast cells, macrophages, and so on (Figure 4A). For immune function, we found that the activity of APC_co_stimulation, APC_co_inhibition, check-point, CCR, parainflammation, MHC_class_I, and type_I_IFN_response were upregulated in patients with high FADD expression (Figure 4B).

### The effect of FADD on immunotherapy and drug sensitivity in lung cancer

**FIGURE 5:**
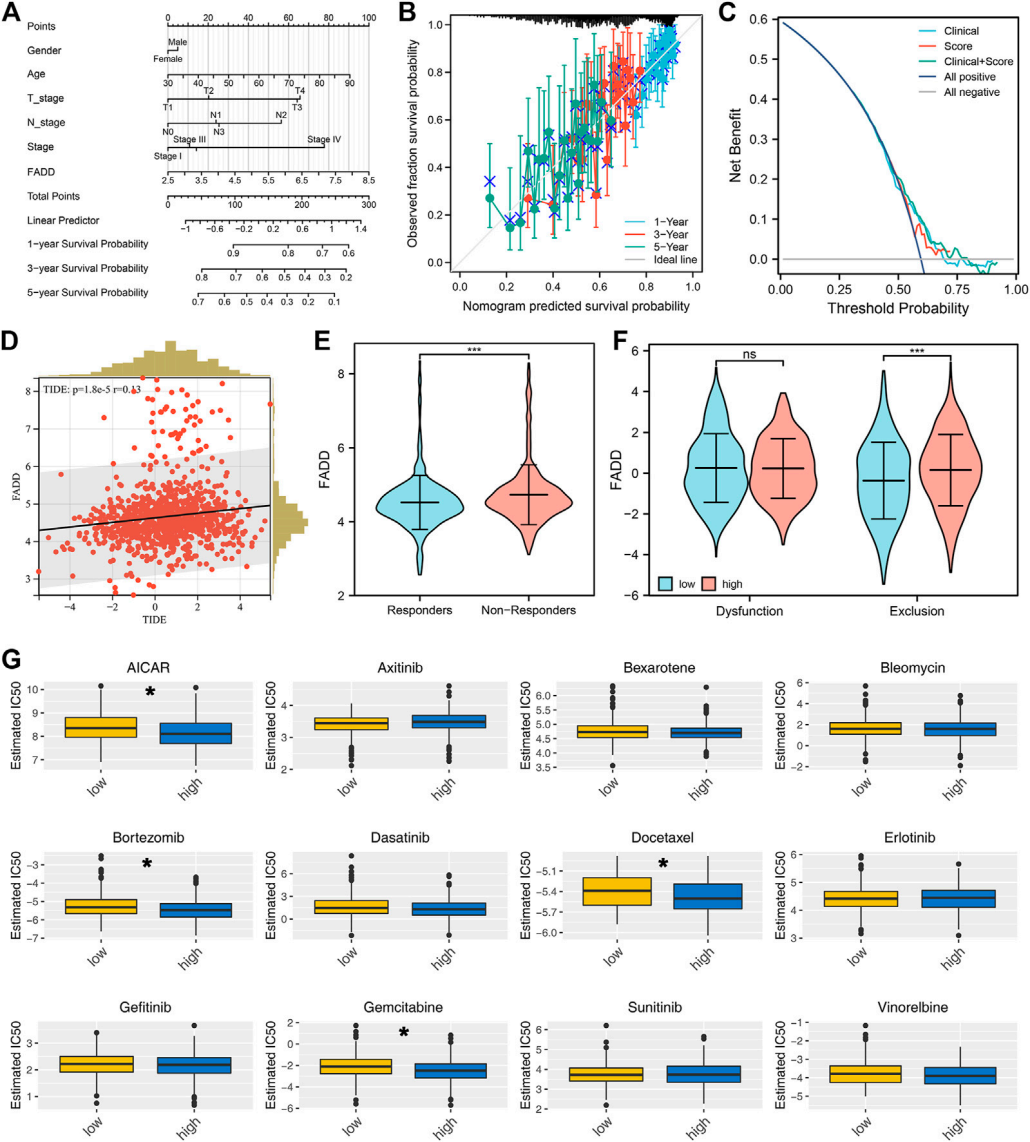
Immunotherapy and drug sensitivity of FADD. Notes: **(A)** A nomogram plot was constructed based on clinical features and FADD; **(B)** Calibration curves; **(C)** DCA curves; **(D)** Correlation between TIDE score and FADD; **(E)** The expression level of FADD in immunotherapy responders and non-responders; **(F)** The immune dysfunction and exclusion in high- and low-FADD expression patients; **(G)** The difference in drug sensitivity among high- and low-FADD expression patients.

Then, we construct a nomogram combining the clinical features and FADD for better prognosis prediction efficiency (Figure 5A). A good agreement was found between observed and anticipated survival times using calibration curves based on the nomogram (Figure 5B). DCA curves showed that combining clinical features could improve the prediction ability of FADD on patient survival (Figure 5C). Immunotherapy is a vital therapy choice for lung cancer. A positive correlation was noticed between the FADD and TIDE score, indicating that patients with high FADD expression might respond worse to immunotherapy (Figure 5D). Meanwhile, we found that the non-responders could have a higher FADD expression (Figure 5E). Moreover, we found that those patients who expressed FADD at a higher level may have a stronger immune exclusion (Figure 5F). High-FADD patients may respond better to AICAR, bortezomib, docetaxel, and gemcitabine, as per the drug sensitivity analysis (Figure 5G).

### FADD promotes the proliferation ability of lung cancer cells

**FIGURE 6:**
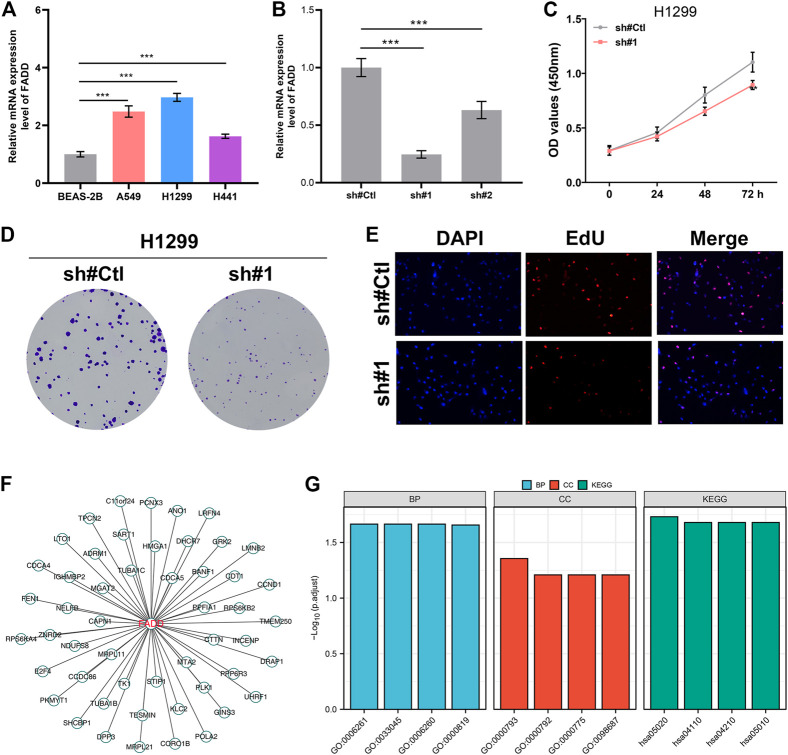
FADD enhances the cell proliferation ability of lung cancer. Notes: **(A)** The mRNA level of FADD in cell lines; **(B)** Knockdown efficiency of FADD in lung cancer cells; **(C)** CCK8 assay was conducted between the control and FADD knockdown cells; **(D)** Colony formation assay was conducted between the control and FADD knockdown cells; **(E)** EdU assay was performed between the control and FADD knockdown cells; **(F)** The molecules regulated by FADD; **(G)** GO and KEGG analysis of FADD-regulated genes.

We next evaluated the biological role of FADD through experiments. The results from qRT-PCR depicted that the FADD was overexpressed in the lung cancer cells (Figure 6A). Following qRT-PCR verification of knockdown efficiency, sh#1 was chosen for further experiments (Figure 6B). Inhibiting FADD reduced lung cancer cell proliferation, as assessed by CCK8 and colony formation assays (Figures 6C, D). EdU assay also demonstrated a lesser percentage of EdU-positive cells in the cells with FADD knockdown (Figure 6E). We next identified the molecules regulated by FADD with |Cor| > 0.3 and *p* < 0.05, defined as FADD-related regulatory molecules (Figure 6F). Biological enrichment assessment depicted that FADD-regulated genes were primarily enriched in the replication of DNA that is DNA-dependent (GO:0006261), regulation of sister chromatid segregation (GO:0033045), DNA replication (GO:0006260), sister chromatid segregation (GO:0000819), condensed chromosome (GO:0000793), heterochromatin (GO:0000792), centromeric region (GO:0000775), chromosome, chromosomal region (GO:0098687), Prion disease (hsa05020), Cell cycle (hsa04110), apoptosis (hsa04210) and Alzheimer disease (hsa05010) (Figure 6G).

### Knockdown of FADD promotes the apoptosis and pyroptosis

**FIGURE 7:**
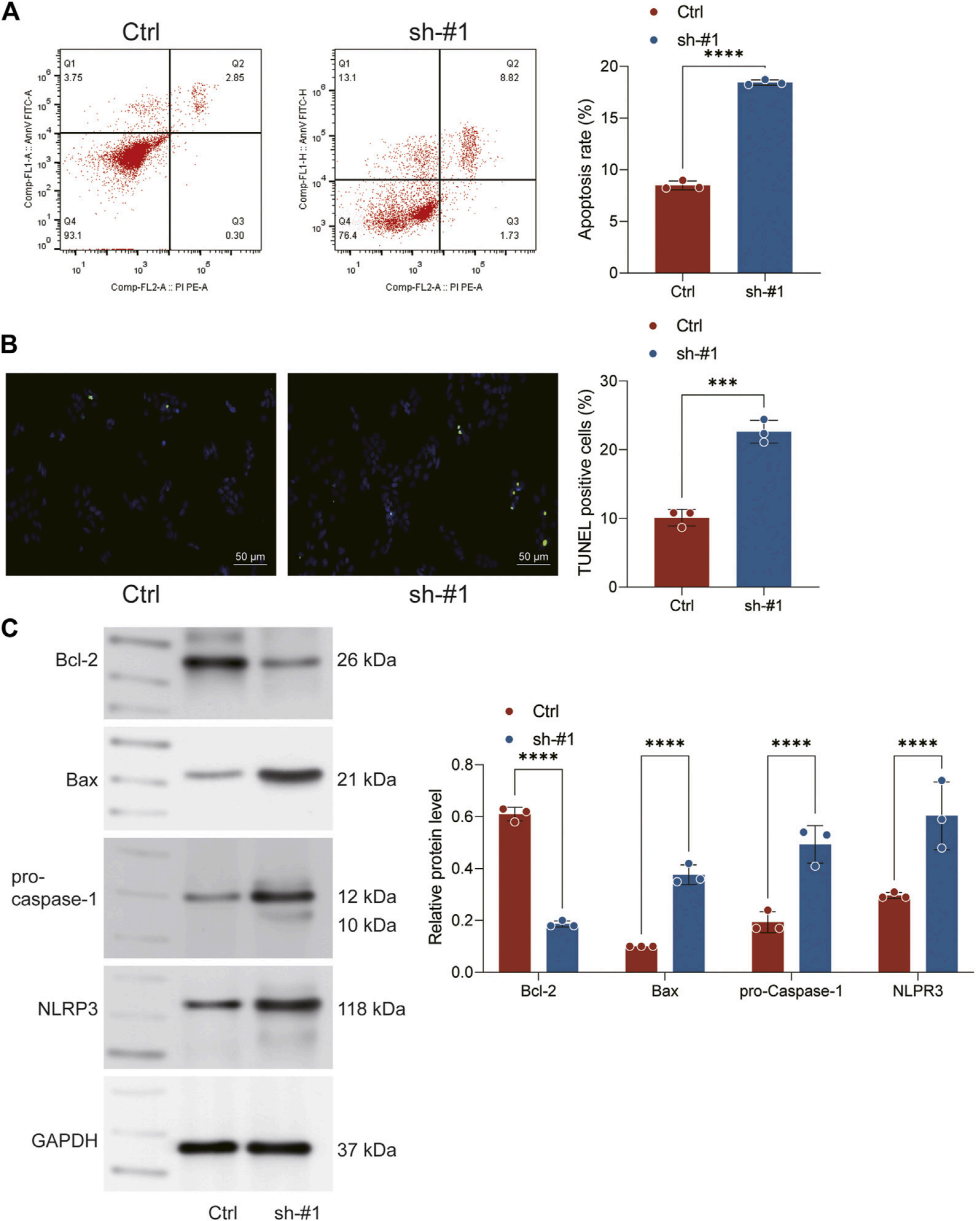
Knockown of FADD promotes the apoptosis and pyroptosis. Notes: **(A)** Flow cytometry was performed to detect cell apoptosis between sh-FADD and control cells; **(B)** TUNEL staining was performed between sh-FADD and control cells; **(C)** Western blot was performed to detect the protein level of specific markers involved in apoptosis and pyroptosis.

The result of flow cytometry showed a higher apoptosis ratio of H1299 cells in sh-FADD cells compared to the control cells, indicating that the knockdown of FADD could promote apoptosis process of lung cancer cells (Figure 7A). The result of TUNEL staining also showed that the same result (Figure 7B). Subsequently, we further used Western blot assay to detect the expression level of apoptosis-related markers (Bcl-2, Bax) and pyroptosis markers (Caspase-1, NLRP3) in H1299 cells. Result indicated that the expression level of Bcl-2 decreased significantly, while the expression level of Bax, Caspase-1 and NLRP3 increased significantly in FADD knockdown cells, indicating that the inhibition of FADD could promote the apoptosis and pyroptosis (Figure 7C).

### Identification of prognosis signature

**FIGURE 8:**
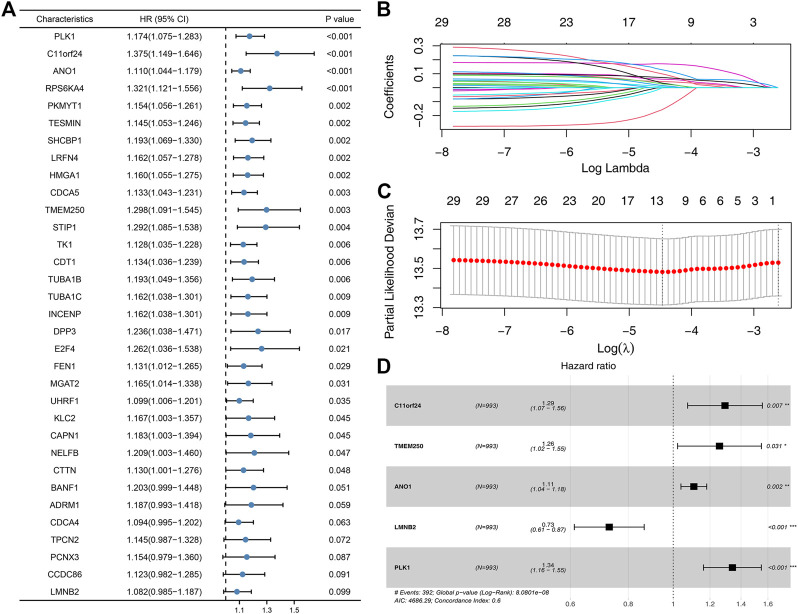
Identification of the prognosis signature based on the FADD-regulated genes. Notes: **(A)** Univariate Cox regression based on the FADD-regulated genes; (**B-C)**: LASSO regression; **(D)** Multivariate Cox regression based on the genes identified by LASSO regression.

**FIGURE 9:**
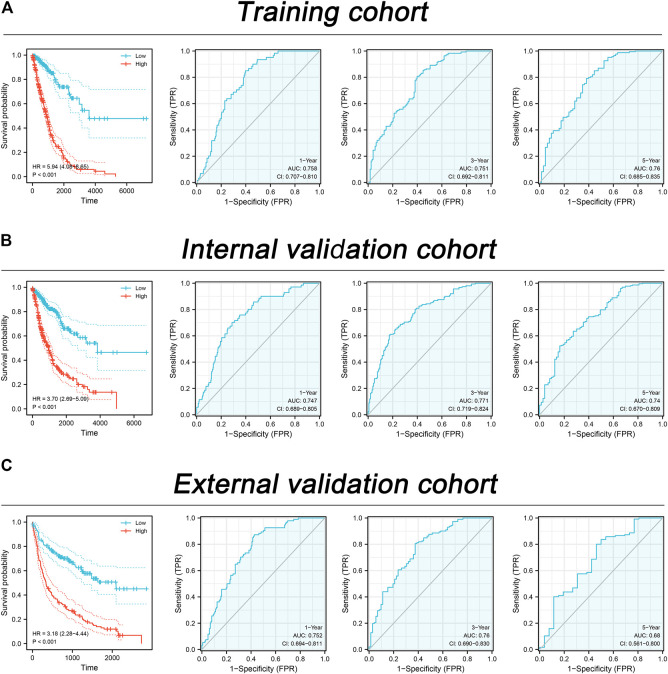
Assessment of the prognosis signature in lung cancer. Notes: **(A)** The performance of our signature in the training cohort; **(B)** The performance of our signature in the internal validation cohort; **(C)** The performance of our signature in the external validation cohort.

Based on the molecule regulated by FADD, we attempted to identify a prognosis signature effectively depicting the patient’s prognosis. Univariate Cox regression was first utilized to determine the prognosis-related genes (Figure 8A, *p* < 0.1). Then, LASSO regression was used for data dimension reduction and optimized variable screening (Figures 8B, C). Ultimately, multivariate Cox regression unraveled a prognosis signature consisting of five genes, C11orf24, TMEM250, ANO1, LMNB2, and PLK1 (Figure 8D). The risk score was evaluated with the equation below: Risk score = C11orf24 * 0.256 + TMEM250 * 0.229 + ANO1 * 0.102 + LMNB2 * −0.317 + PLK1 * 0.294 (Figure 8D). The result indicated that our signature performed well in the training cohort. The KM survival curve showed that the high-risk patients might have a worse OS (Figure 9A). The AUC values over 1, 3, and 5 years were 0.758, 0.751, and 0.76, respectively (Figure 9A). The same result was also observed in the internal validation as well as external validation cohorts (Figures 9B, C, internal validation cohort: 1, 3, and 5 years were 0.747, 0.771, and 0.74, respectively; external validation cohort: 1, 3, and 5 years were 0.752, 0.76, and 0.68, respectively).

## Discussion

There is a growing concern that lung cancer will pose a huge threat to human health and will further exacerbate the world’s already high cancer rate (Sung et al., 2021; Pan et al., 2022b). Despite tremendous human efforts in early prevention, early diagnosis and treatment, and individualized treatment of lung cancer, millions of lung cancer patients still die worldwide each year. Therefore, more analyses of the specific causes of lung cancer and the identification of novel therapeutic targets aimed at managing this disease remain crucial (Wang et al., 2021).

In our study, we collected the PANoptosis-related genes from previous studies. Through series analysis, we identified the FADD, an adaptor of PANoptosis and apoptosis, for further analysis. Results showed that FADD is a potential risk factor for lung cancer and is mainly localized in nucleoplasm and cytosol. We next performed immune infiltration analysis (IIA) and biological enrichment to illustrate the fundamental effects of FADD on lung cancer. Subsequently, we discovered that the patients with a high level of FADD might respond worse to immunotherapy but better to AICAR, bortezomib, docetaxel, and gemcitabine. *In vitro* experiments indicated that FADD inhibition could remarkably suppress the proliferation ability of lung cancer tissues. Ultimately, a prognosis signature was identified based on the FADD-regulated genes, which showed satisfactory prediction efficiency on patients’ OS in training, as well as internal validation and external validation cohorts.

Firstly, we identified five PANoptosis-related genes closely related to patients’ survival, including FADD, TNFRSF1A, CASP9, MLKL, and CASP4. These genes have been reported to be involved in cancer development. For instance, in breast cancer, Egusquiaguirre et al. found that the TNFRSF1A was a target gene of STAT3 and could affect cancer development through NF-κB signaling (Egusquiaguirre et al., 2018). Wang et al. revealed that the TNFRSF1A might exert as a prognosis and immune infiltration biomarker for the glioblastoma multiforme (Wang et al., 2022b). Using a cellular mechanism that involves targeting CASP9 and enhancing the ubiquitination degradation of p53, Jing et al. observed the exosome-transmitted miR-769-5p imparts cisplatin resistance and gastric cancer progression (Jing et al., 2022). Li et al. revealed that LINC00607 could facilitate thyroid cancer progression by regulating the CASP9 promoter methylation (Li et al., 2021). Han et al. indicated that the HSP90 could induce drug resistance in NSCLC (Han et al., 2018). Wang et al., the upregulated RIP3 alleviates the progression of prostate cancer through activating RIP3/MLKL signaling pathway and inducting cell necroptosis (Wang et al., 2020). Wang et al. showed that necroptosis could regulate tumor repopulation after radiotherapy through RIP1/RIP3/MLKL/JNK/IL8 pathway (Wang et al., 2019b). Our result identified the FADD, TNFRSF1A, CASP9, MLKL, and CASP4 involved in the PANoptosis process and significantly affect patient survival, which can provide future direction in cancer research.

Biological enrichment analysis depicted that the pathways of glycolysis, mitotic spindle, inflammatory response, mTORC1 signaling, TNFA signaling *via* NFKB, apical junction, G2M check-point, interferon-gamma response, EMT, and E2F target were activated high FADD expression patients. Glycolysis has been reported to participate in the progression course of lung cancer. Lin et al. observed that fascin could enhance lung cancer growth and metastasis by upregulating glycolysis activity (Lin et al., 2021). Zhou et al. revealed that glycolysis could be promoted by circRNA-ENO1 and lung cancer development by upregulating the host genes ENO1 (Zhou et al., 2019). Moreover, Reddy et al. indicated that lanatoside C could induce G2/M cell cycle arrest and restrict cancer cell proliferation by attenuating MAPK, JAK-STAT, Wnt, and PI3K/AKT/mTOR signaling (Reddy et al., 2019). Almasi et al. found that in lung cancer tissues, the inhibition of TRPM2 could lead to G2/M arrest and apoptosis by increasing intracellular RNS and ROS levels and activating the JNK pathway (Almasi et al., 2019). Chae et al. also revealed that the EMT signature could remarkably affect the T-cell infiltration in the lung cancer microenvironment (Chae et al., 2018). Our result indicated that the cancer-promoting role of FADD in lung cancer might be exerted by regulating the above pathways.

The immune-related analysis also indicated that FADD could affect many immune cells in the lung cancer microenvironment. Meanwhile, we noticed that the patients with high FADD expression might respond worse to immunotherapy. Zhang et al. found that the CCL7 can recruit cDC1 to enhance anti-tumor immunity and check-point immunotherapy of lung cancer (Zhang et al., 2020). Moreover, Dai et al. indicated that the USP7 targeting could modulate the anti-tumor immune response as a result of the reprogramming of tumor-associated macrophages in lung cancer (Dai et al., 2020). Our results indicated that FADD might also be an immune-related gene of lung cancer.

Although our research is based on high-quality analysis, some limitations still need to be noted. Firstly, the samples we analyzed mostly come from Western populations. In fact, significant biological differences exist between different races. It is hard for our study to avoid the underlying race bias. Secondly, the conclusion of our result was based on the levels of mRNA but not the protein. The existence of post-transcriptional modification will reduce the credibility of our research.

## Data Availability

The original contributions presented in the study are included in the article/Supplementary Material, further inquiries can be directed to the corresponding authors.

## Supplementary material

Supplementary Figure S1 The flow chart of whole study.

Supplementary Figure S2 The ROC curve of FADD on OS.

Supplementary Figure S3 The FADD expression pattern of the pan-cancer.

## References

[B1] Aberle D. R. Adams A. M. Berg C. D. Black W. C. Clapp J. D. Fagerstrom R. M. (2011). Reduced lung-cancer mortality with low-dose computed tomographic screening. N. Engl. J. Med. 365 (5), 395–409. 10.1056/NEJMoa1102873 21714641 PMC4356534

[B2] Almasi S. Long C. Y. Sterea A. Clements D. R. Gujar S. El Hiani Y. (2019). TRPM2 silencing causes G2/M arrest and apoptosis in lung cancer cells via increasing intracellular ROS and RNS levels and activating the JNK pathway. Cell. physiology Biochem. Int. J. Exp. Cell. physiology, Biochem. Pharmacol. 52 (4), 742–757. 10.33594/000000052 30933439

[B3] Chae Y. K. Chang S. Ko T. Anker J. Agte S. Iams W. (2018). Epithelial-mesenchymal transition (EMT) signature is inversely associated with T-cell infiltration in non-small cell lung cancer (NSCLC). Sci. Rep. 8 (1), 2918. 10.1038/s41598-018-21061-1 29440769 PMC5811447

[B4] Chen B. Khodadoust M. S. Liu C. L. Newman A. M. Alizadeh A. A. (2018). Profiling tumor infiltrating immune cells with CIBERSORT. Methods Mol. Biol. Clift. NJ) 1711, 243–259. 10.1007/978-1-4939-7493-1_12 PMC5895181 29344893

[B5] Dai X. Lu L. Deng S. Meng J. Wan C. Huang J. (2020). USP7 targeting modulates anti-tumor immune response by reprogramming Tumor-associated Macrophages in Lung Cancer. Theranostics 10 (20), 9332–9347. 10.7150/thno.47137 32802195 PMC7415808

[B6] Egusquiaguirre S. P. Yeh J. E. Walker S. R. Liu S. Frank D. A. (2018). The STAT3 target gene TNFRSF1A modulates the NF-κB pathway in breast cancer cells. Neoplasia (New York, NY) 20 (5), 489–498. 10.1016/j.neo.2018.03.004 PMC5916089 29621649

[B7] Fu J. Li K. Zhang W. Wan C. Zhang J. Jiang P. (2020). Large-scale public data reuse to model immunotherapy response and resistance. Genome Med. 12 (1), 21. 10.1186/s13073-020-0721-z 32102694 PMC7045518

[B8] Gu X. Wei S. Li Z. Xu H. (2022). Machine learning reveals two heterogeneous subtypes to assist immune therapy based on lipid metabolism in lung adenocarcinoma. Front. Immunol. 13, 1022149. 10.3389/fimmu.2022.1022149 36238302 PMC9551187

[B9] Han J. Goldstein L. A. Hou W. Chatterjee S. Burns T. F. Rabinowich H. (2018). HSP90 inhibition targets autophagy and induces a CASP9-dependent resistance mechanism in NSCLC. Autophagy 14 (6), 958–971. 10.1080/15548627.2018.1434471 29561705 PMC6103412

[B10] Jing X. Xie M. Ding K. Xu T. Fang Y. Ma P. (2022). Exosome-transmitted miR-769-5p confers cisplatin resistance and progression in gastric cancer by targeting CASP9 and promoting the ubiquitination degradation of p53. Clin. Transl. Med. 12 (5), e780. 10.1002/ctm2.780 35522909 PMC9076018

[B11] Karki R. Lee S. Mall R. Pandian N. Wang Y. Sharma B. R. (2022). ZBP1-dependent inflammatory cell death, PANoptosis, and cytokine storm disrupt IFN therapeutic efficacy during coronavirus infection. Sci. Immunol. 7 (74), eabo6294. 10.1126/sciimmunol.abo6294 35587515 PMC9161373

[B12] Karki R. Sundaram B. Sharma B. R. Lee S. Malireddi R. K. S. Nguyen L. N. (2021). ADAR1 restricts ZBP1-mediated immune response and PANoptosis to promote tumorigenesis. Cell Rep. 37 (3), 109858. 10.1016/j.celrep.2021.109858 34686350 PMC8853634

[B13] Lee S. Karki R. Wang Y. Nguyen L. N. Kalathur R. C. Kanneganti T. D. (2021). AIM2 forms a complex with pyrin and ZBP1 to drive PANoptosis and host defence. Nature 597 (7876), 415–419. 10.1038/s41586-021-03875-8 34471287 PMC8603942

[B14] Li L. Gao Z. Zhao L. Ren P. Shen H. (2021). Long non-coding RNA LINC00607 silencing exerts antioncogenic effects on thyroid cancer through the CASP9 Promoter methylation. J. Cell. Mol. Med. 25 (16), 7608–7620. 10.1111/jcmm.16265 34232553 PMC8358880

[B15] Li S. Cong X. Gao H. Lan X. Li Z. Wang W. (2019). Tumor-associated neutrophils induce EMT by IL-17a to promote migration and invasion in gastric cancer cells. J. Exp. Clin. cancer Res. CR 38 (1), 6. 10.1186/s13046-018-1003-0 30616627 PMC6323742

[B16] Lin J. F. Hu P. S. Wang Y. Y. Tan Y. T. Yu K. Liao K. (2022). Phosphorylated NFS1 weakens oxaliplatin-based chemosensitivity of colorectal cancer by preventing PANoptosis. Signal Transduct. Target Ther. 7 (1), 54. 10.1038/s41392-022-00889-0 35221331 PMC8882671

[B17] Lin S. Li Y. Wang D. Huang C. Marino D. Bollt O. (2021). Fascin promotes lung cancer growth and metastasis by enhancing glycolysis and PFKFB3 expression. Cancer Lett. 518, 230–242. 10.1016/j.canlet.2021.07.025 34303764 PMC8355190

[B18] Malireddi R. K. S. Karki R. Sundaram B. Kancharana B. Lee S. Samir P. (2021). Inflammatory cell death, PANoptosis, mediated by cytokines in diverse cancer lineages inhibits tumor growth. ImmunoHorizons 5 (7), 568–580. 10.4049/immunohorizons.2100059 34290111 PMC8522052

[B19] Molina J. R. Yang P. Cassivi S. D. Schild S. E. Adjei A. A. (2008). Non-small cell lung cancer: Epidemiology, risk factors, treatment, and survivorship. Mayo Clin. Proc. 83 (5), 584–594. 10.4065/83.5.584 18452692 PMC2718421

[B20] Pan H. Pan J. Li P. Gao J. (2022). Characterization of PANoptosis patterns predicts survival and immunotherapy response in gastric cancer. Clin. Immunol. 238, 109019. 10.1016/j.clim.2022.109019 35470064

[B21] Pan S. Sun S. Liu B. Hou Y. (2022). Pan-cancer landscape of the RUNX protein family reveals their potential as carcinogenic biomarkers and the mechanisms underlying their action. J. Transl. Intern. Med. 10 (2), 156–174. 10.2478/jtim-2022-0013 PMC9328034 35959452

[B22] Plattner C. Finotello F. Rieder D. (2020). Deconvoluting tumor-infiltrating immune cells from RNA-seq data using quanTIseq. Methods Enzym. 636, 261–285. 10.1016/bs.mie.2019.05.056 32178821

[B23] Racle J. Gfeller D. (2020). Epic: A tool to estimate the proportions of different cell types from bulk gene expression data. Methods Mol. Biol. Clift. NJ) 2120, 233–248. 10.1007/978-1-0716-0327-7_17 32124324

[B24] Reck M. Remon J. Hellmann M. D. (2022). First-line immunotherapy for non-small-cell lung cancer. J. Clin. Oncol. 40 (6), 586–597. 10.1200/JCO.21.01497 34985920

[B25] Reddy D. Kumavath R. Ghosh P. Barh D. (2019). Lanatoside C induces G2/M cell cycle arrest and suppresses cancer cell growth by attenuating MAPK, Wnt, JAK-STAT, and PI3K/AKT/mTOR signaling pathways. Biomolecules 9 (12), 792. 10.3390/biom9120792 31783627 PMC6995510

[B26] Ritchie M. E. Phipson B. Wu D. Hu Y. Law C. W. Shi W. (2015). Limma powers differential expression analyses for RNA-sequencing and microarray studies. Nucleic acids Res. 43 (7), e47. 10.1093/nar/gkv007 25605792 PMC4402510

[B27] Shannon P. Markiel A. Ozier O. Baliga N. S. Wang J. T. Ramage D. (2003). Cytoscape: A software environment for integrated models of biomolecular interaction networks. Genome Res. 13 (11), 2498–2504. 10.1101/gr.1239303 14597658 PMC403769

[B28] Subramanian A. Tamayo P. Mootha V. K. Mukherjee S. Ebert B. L. Gillette M. A. (2005). Gene set enrichment analysis: A knowledge-based approach for interpreting genome-wide expression profiles. Proc. Natl. Acad. Sci. U. S. A. 102 (43), 15545–15550. 10.1073/pnas.0506580102 16199517 PMC1239896

[B29] Sung H. Ferlay J. Siegel R. L. Laversanne M. Soerjomataram I. Jemal A. (2021). Global cancer statistics 2020: GLOBOCAN estimates of incidence and mortality worldwide for 36 cancers in 185 countries. CA Cancer J. Clin. 71 (3), 209–249. 10.3322/caac.21660 33538338

[B30] Tan A. C. Tan D. S. W. (2022). Targeted therapies for lung cancer patients with oncogenic driver molecular alterations. J. Clin. Oncol. 40 (6), 611–625. 10.1200/JCO.21.01626 34985916

[B31] Wang K. J. Wang K. Y. Zhang H. Z. Meng X. Y. Chen J. F. Wang P. (2020). Up-regulation of RIP3 alleviates prostate cancer progression by activation of RIP3/MLKL signaling pathway and induction of necroptosis. Front. Oncol. 10, 1720. 10.3389/fonc.2020.01720 32984054 PMC7480187

[B32] Wang W. Green M. Choi J. E. Gijon M. Kennedy P. D. Johnson J. K. (2019). CD8(+) T cells regulate tumour ferroptosis during cancer immunotherapy. Nature 569 (7755), 270–274. 10.1038/s41586-019-1170-y 31043744 PMC6533917

[B33] Wang X. Yang G. Wang Q. Zhao Y. Ding K. Ji C. (2022). C1R, CCL2, and TNFRSF1A genes in coronavirus disease-COVID-19 pathway serve as novel molecular biomarkers of GBM prognosis and immune infiltration. Dis. markers 2022, 8602068. 10.1155/2022/8602068 35726234 PMC9206210

[B34] Wang Y. Hou K. Jin Y. Bao B. Tang S. Qi J. (2021). Lung adenocarcinoma-specific three-integrin signature contributes to poor outcomes by metastasis and immune escape pathways. J. Transl. Intern. Med. 9 (4), 249–263. 10.2478/jtim-2021-0046 PMC8802404 35136724

[B35] Wang Y. Kanneganti T. D. (2021). From pyroptosis, apoptosis and necroptosis to PANoptosis: A mechanistic compendium of programmed cell death pathways. Comput. Struct. Biotechnol. J. 19, 4641–4657. 10.1016/j.csbj.2021.07.038 34504660 PMC8405902

[B36] Wang Y. Pandian N. Han J. H. Sundaram B. Lee S. Karki R. (2022). Single cell analysis of PANoptosome cell death complexes through an expansion microscopy method. Cell. Mol. life Sci. CMLS 79 (10), 531. 10.1007/s00018-022-04564-z 36169732 PMC9545391

[B37] Wang Y. Zhao M. He S. Luo Y. Cheng J. Gong Y. (2019). Necroptosis regulates tumor repopulation after radiotherapy via RIP1/RIP3/MLKL/JNK/IL8 pathway. J. Exp. Clin. cancer Res. CR 38 (1), 461. 10.1186/s13046-019-1423-5 31706322 PMC6842489

[B38] Wei Y. Chen X. Ren X. Wang B. Zhang Q. Bu H. (2021). Identification of MX2 as a novel prognostic biomarker for sunitinib resistance in clear cell renal cell carcinoma. Front. Genet. 12, 680369. 10.3389/fgene.2021.680369 34306023 PMC8299280

[B39] Wu D. Yin Z. Ji Y. Li L. Li Y. Meng F. (2021). Identification of novel autophagy-related lncRNAs associated with a poor prognosis of colon adenocarcinoma through bioinformatics analysis. Sci. Rep. 11 (1), 8069. 10.1038/s41598-021-87540-0 33850225 PMC8044244

[B40] Yang W. Soares J. Greninger P. Edelman E. J. Lightfoot H. Forbes S. (2013). Genomics of drug sensitivity in cancer (GDSC): A resource for therapeutic biomarker discovery in cancer cells. Nucleic acids Res. 41, D955–D961. 10.1093/nar/gks1111 23180760 PMC3531057

[B41] Yin Z. Wu D. Shi J. Wei X. Jin N. Lu X. (2020). Identification of ALDH3A2 as a novel prognostic biomarker in gastric adenocarcinoma using integrated bioinformatics analysis. BMC cancer 20 (1), 1062. 10.1186/s12885-020-07493-x 33148208 PMC7640415

[B42] Yu G. Wang L. G. Han Y. He Q. Y. (2012). clusterProfiler: an R package for comparing biological themes among gene clusters. Omics a J. Integr. Biol. 16 (5), 284–287. 10.1089/omi.2011.0118 PMC3339379 22455463

[B43] Zhang M. Yang W. Wang P. Deng Y. Dong Y. T. Liu F. F. (2020). CCL7 recruits cDC1 to promote antitumor immunity and facilitate checkpoint immunotherapy to non-small cell lung cancer. Nat. Commun. 11 (1), 6119. 10.1038/s41467-020-19973-6 33257678 PMC7704643

[B44] Zhou J. Zhang S. Chen Z. He Z. Xu Y. Li Z. (2019). CircRNA-ENO1 promoted glycolysis and tumor progression in lung adenocarcinoma through upregulating its host gene ENO1. Cell death Dis. 10 (12), 885. 10.1038/s41419-019-2127-7 31767835 PMC6877563

