# Supplementary figures and images for: Comprehensive analysis illustrating the role of PANoptosis-related genes in lung cancer based on bioinformatic algorithms and experiments
Source: Front Pharmacol. 2023 Feb 16;14:1115221. doi: 10.3389/fphar.2023.1115221 (PMC9977813; doi:10.3389/fphar.2023.1115221)

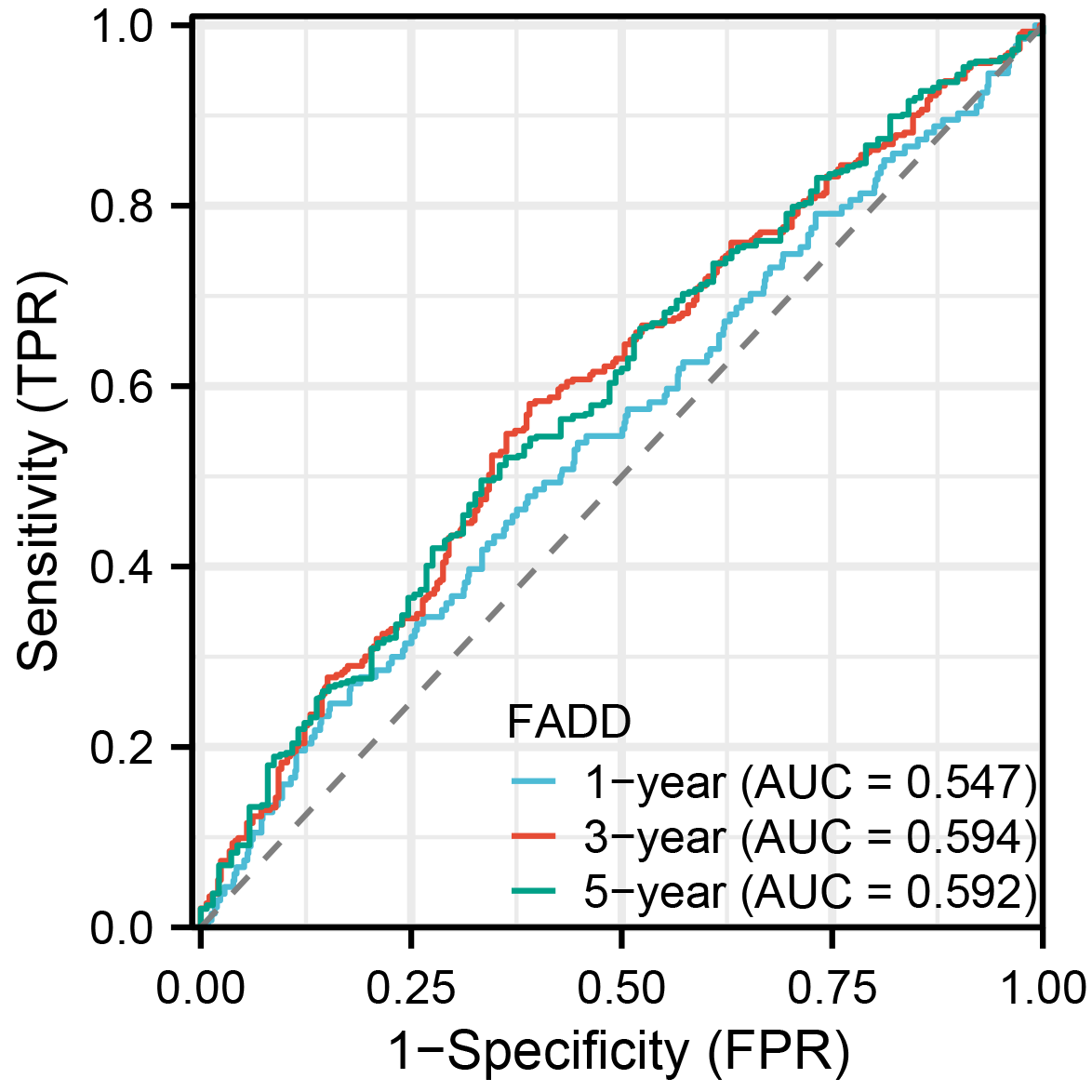

Supplement: Supplementary file 3 [file Image2.TIF]

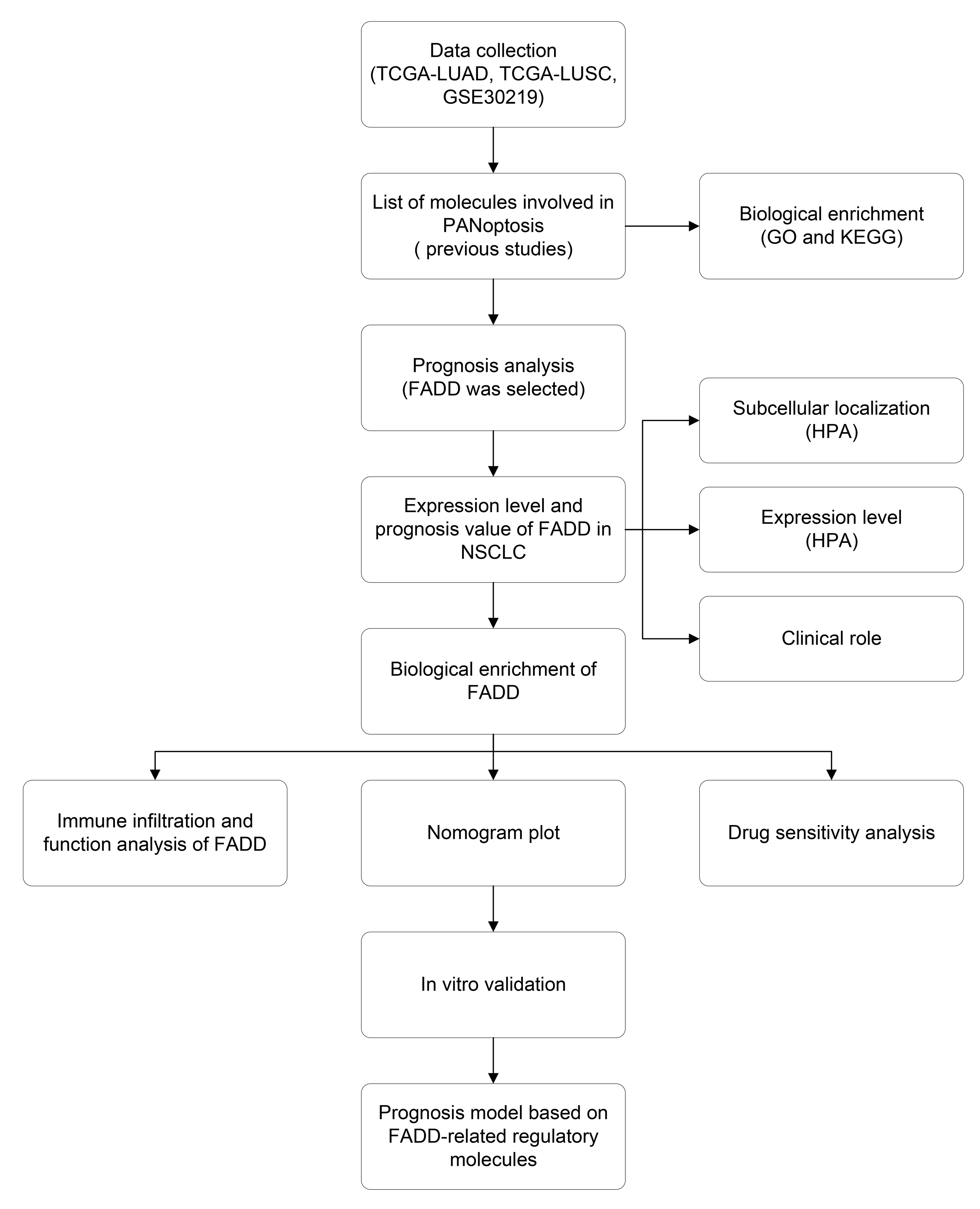

Supplement: Supplementary file 4 [file Image1.TIF]
